# Comparison of Boiling and Robotics Automation Method in DNA Extraction for Metagenomic Sequencing of Human Oral Microbes

**DOI:** 10.1371/journal.pone.0154389

**Published:** 2016-04-22

**Authors:** Junya Yamagishi, Yukuto Sato, Natsuko Shinozaki, Bin Ye, Akito Tsuboi, Masao Nagasaki, Riu Yamashita

**Affiliations:** 1 Division of Collaboration and Education, Research Center for Zoonosis Control, Hokkaido University, North 20, West 10 Kita-ku, Sapporo, Hokkaido, 001–0020, Japan; 2 Global Station for Zoonosis Control, GI-CoRE, Hokkaido University, North 20, West 10 Kita-ku, Sapporo, Hokkaido, 001–0020, Japan; 3 Department of Integrative Genomics, Tohoku Medical Megabank Organization, Tohoku University, 2–1, Seiryo-machi, Aoba-ku, Sendai, Miyagi, 980–8573, Japan; 4 Department of Community Medical Supports, Tohoku Medical Megabank Organization, Tohoku University, 2–1, Seiryo-machi, Aoba-ku, Sendai, Miyagi, 980–8573, Japan; Medical University of South Carolina, UNITED STATES

## Abstract

The rapid improvement of next-generation sequencing performance now enables us to analyze huge sample sets with more than ten thousand specimens. However, DNA extraction can still be a limiting step in such metagenomic approaches. In this study, we analyzed human oral microbes to compare the performance of three DNA extraction methods: PowerSoil (a method widely used in this field), QIAsymphony (a robotics method), and a simple boiling method. Dental plaque was initially collected from three volunteers in the pilot study and then expanded to 12 volunteers in the follow-up study. Bacterial flora was estimated by sequencing the V4 region of 16S rRNA following species-level profiling. Our results indicate that the efficiency of PowerSoil and QIAsymphony was comparable to the boiling method. Therefore, the boiling method may be a promising alternative because of its simplicity, cost effectiveness, and short handling time. Moreover, this method was reliable for estimating bacterial species and could be used in the future to examine the correlation between oral flora and health status. Despite this, differences in the efficiency of DNA extraction for various bacterial species were observed among the three methods. Based on these findings, there is no “gold standard” for DNA extraction. In future, we suggest that the DNA extraction method should be selected on a case-by-case basis considering the aims and specimens of the study.

## Introduction

A large variety and number of microbes can coexist with animals, including humans [1]. For example, it is estimated that 100 trillion cells consisting of 300–500 microbe species exist in the human gut, and 700 microbe species are found in the human oral cavity [2, 3]. These microbes can have a profound impact on human health. Indeed, correlations between diseases (e.g., obesity, metabolic syndrome, type 1 diabetes, type 2 diabetes, autism disorder, and rheumatic diseases) and the microbiome have been demonstrated over the last 10 years [4–8]. Such correlations have been feasible because of advances in next-generation sequencing (NGS), which allows exhaustive and populational analysis without cultivation of individual microbes.

DNA extraction is one of the key steps for metagenomic analysis by NGS. For DNA extraction, the purity, yield, level of contamination, and scalability (including automation and cost) must be considered. In addition, low bias is desirable for DNA extraction from diverse flora. To date, silica column based methods, such as PowerSoil (MO Bio Laboratories) [9] or the QIAamp DNA Stool Mini Kit (QIAGEN) [9–12], are widely applied. Similarly, QIAsymphony system (QIAGEN) has protocols for automated DNA extraction for microbes. Alternatively, easy handling methods, such as Chelex [13] or boiling methods [12], may have potential advantages for sample collection in large cohort studies.

A series of studies have evaluated the performance of these different DNA extraction methods [9–12, 14–16]. Some reports indicate that the addition of a bead beating step can contribute to increased DNA yield and comprehensiveness [9–11]. In addition, enzymatic treatment (e.g., with mutanolysin) also increases the performance [9]. Nevertheless, an optimal standard method for sample preparation for use in NGS has not been determined. One factor that may account for the lack of a standard DNA extraction method is that some of the previous studies did not use the most recent NGS methods [11, 14]. However, perhaps the more important reason is that it is impossible to validate these methods simply by comparing bacterial flora, as the environmental flora is unknown [10–12, 14]. In contrast, the artificial flora, consisting of dozens of given microbes within a population can be evaluated; however, the observation should be limited to the microbes examined [9, 15]. Therefore, it is difficult to test the bias of these DNA extraction methods because it is virtually impossible to examine every microbe with infinite diversity.

The oral microbiome is related to carious or periodontal disease, and more recently has been linked to systemic diseases, such as atherosclerotic cardiovascular disease [17] and Alzheimer disease [18], and also to preterm births [19]. Therefore, the analysis of oral samples is important to understand the relationship between human health and microbiota.

The aim of this study was to perform a comparative analysis of typical DNA extraction methods (including PowerSoil, semi-modified automated QIAsymphony, and simple boiling) on human oral samples. The methods were validated with 16S ribosomal DNA sequencing. Our observations suggest no major drawbacks among the three methods in terms of bias; however, small differences in DNA extraction efficiency were observed for some bacterial species.

## Materials and Methods

### Ethics Statemetnt

All relevant research protocols and procedures were approved by the Ethics Committee of Tohoku University School of Medicine, Sendai, Japan. All adult subjects provided written informed consent.

### Subjects and sampling

In this study, we collected two sets of specimen, oral plaque samples provided from three healthy Japanese male volunteers (A–C) for proof-of-concept initial evaluation and following 12 samples for optimization and detailed evaluation.

In the former case, subjects were confirmed by a dentist to have no severe caries, periodontal disease, or any other dental disorders. The ages of the subjects ranged from 35 to 41 years. The Decayed-Missing-Filled Teeth (DMFT) index of the subjects was 7.7, including wisdom teeth (max = 32). The detailed clinical data at the time of sampling was as follows: volunteer A, DMFT = 14 (D = 1, M = 0, F = 13), number of teeth = 29 (number of wisdom teeth = 1, one wisdom tooth restored), and active caries = 1; volunteer B, DMFT = 9 (D = 0, M = 0, F = 9), number of teeth = 29 (number of wisdom teeth = 1, one wisdom tooth restored), and active caries = 0; volunteer C, DMFT = 0 (D = 0, M = 0, F = 0), number of teeth = 29 (number of wisdom teeth = 2, left lower permanent canine and lateral incisor are fused), and active caries = 0. The supragingival plaques were taken from all teeth by the subjects themselves using a sterilized plastic toothpick and dissolved with 0.5 mL of Tris-EDTA (10 mM Tris and 1 mM EDTA; pH 8.0). Volunteer A provided three independent samples taken over four sequential days.

In the latter case, 12 specimen including the volunteers A to C and additional nine volunteers consist of eight Japanese males and one Japanese female, provided supragingival plaque samples collected in the same manner as described above. The pooled plaque samples were mixed by vortex, subdivided into daughter tubes corresponding to the series of DNA extraction methods, and kept at −30°C until the total DNA was extracted.

### DNA extraction

For the initial evaluation with specimens from three volunteers, the following three kits or methods were used for DNA extraction: the PowerSoil DNA Isolation Kit (MO BIO, Carlsbad, CA, USA), the QIAsymphony SP instrument and QIAsymphony DSP Virus/Pathogen Mini Kit, and the boiling method. DNA extraction with the PowerSoil DNA Isolation Kit was performed according to the manufacturer’s protocol. Extraction using the QIAsymphony SP instrument and the QIAsymphony DSP Virus/Pathogen Mini Kit was executed according to the Complex200_V6_DSP protocol with the following modifications in plaque sample pretreatment: after brief centrifugation of the collected plaque sample (10,000 g for 1 min), pellets were resuspended in 300 μL of Buffer ATL (QIAGEN) containing 2 μL of Reagent DX (QIAGEN) and incubated for 15 min at 56°C (Table 1 “QIAsymphony”). Subsequently, the supernatant was transferred to baskets in the Investigator Lyse&Spin Basket Kit (QIAGEN) and centrifuged (8,000 g for 5 s) (Table 1 “QIAsymphony + filtration”). Alternatively, the supernatant were transferred into a Pathogen Lysis Tube (QIAGEN) and homogenized with the Mixer Mill MM 400 (Retsch, Haan, Germany) for 10 min with a vibrational frequency of 20 Hz, and then filtered with the Investigator Lyse&Spin Basket Kit (Table 1 “QIAsymphony + beads beating + filtration”). For DNA extraction by the boiling method, suspended plaque samples were incubated at 99°C for 15 min, with or without adding 0.5% Tween 20, and immediately cooled on ice (Table 1 “boil” or “boil + tween,” respectively).

**Table 1:** Description of samples used in this study.

sample ID	volunteer	series	method
1	A	1	PowerSoil
2	A	1	boil
3	A	1	boil
4	A	1	boil
5	A	1	boil + tween
6	A	1	boil + tween
7	A	1	boil + tween
8	A	1	QIAsymphony
9	A	1	QIAsymphony + filtration
10	A	1	QIAsymphony + beads beating + filtration
11	A	2	PowerSoil
12	A	2	boil
13	A	2	boil + tween
14	A	2	QIAsymphony
15	A	2	QIAsymphony + filtration
16	A	2	QIAsymphony + beads beating + filtration
17	A	3	PowerSoil
18	A	3	boil
19	A	3	boil + tween
20	B	1	PowerSoil
21	B	1	boil
22	B	1	boil + tween
23	C	1	PowerSoil
24	C	1	boil
25	C	1	boil + tween

For the latter 12 specimen, an optional pretreatment step of bead beating was performed using 0.1 mm glass beads (AZ-ONE, BZ-01) and the Mixer Mill MM 400 for 10 min at 20 Hz. For comparison, we also incubated the plaque samples with 0.5% Tween 20, mutanolysin (0.3 U/μL), and lysostaphin (24 mU/μL) at 37°C for 60 min.

### PCR amplification and amplicon sequencing of bacterial 16S rRNA V4 region

Using the extracted oral plaque DNA as a template, a partial 16S rRNA gene of the bacterial community was amplified by two PCR steps as described in our previous study of the oral microbiome [20]. In brief, a partial sequence of the hypervariable V4 region of the bacterial 16S rRNA gene was amplified by PCR using a primer set that has a conserved sequence in bacterial 16S rRNA and a tag for the second PCR step. For the second PCR step, the first PCR products were amplified with 12 cycles by using primers that contain a sequence against the tag from the first PCR step, a dual-index tag sequence, and flowcell binding sites of the Illumina adapter. The tag-indexed second PCR products obtained above were sequenced with the MiSeq sequencer in a multiplex manner, using a 250 bp paired-end sequencing protocol with the MiSeq sequencing reagent kits v2 (Illumina), according to the manufacturer’s instructions.

### Primary data processing and quality control of amplicon sequences

The obtained raw sequences of bacterial 16S rRNA V4 amplicons were subjected to primary data processing, including low-quality tail base trimming, paired-end read assembly, and primer sequence removal, as described in our previous study [20]. In brief, a total data-quality of each sequencing run was evaluated with FastQC and SUGAR [21], the low-quality tail of each sequence read was trimmed with DynamicTrim [22], the tail-trimmed paired-end reads (forward and reverse) were assembled with FLASH [23], the assembled sequences were filtered by custom Perl scripts to remove reads containing N-bases or having abnormal sequence length, and then the remaining head- and tail-sequences originating from the primers were removed with TagCleaner [24].

### Taxonomic assignments and comparative analyses among the DNA extraction methods

The assembled amplicon sequences were applied to sequence similarity-based taxonomic assignments to conduct species composition and comparative diversity analyses of oral bacteria based on our previous study [20]. In brief, redundant sequences in a data set were merged into a single sequence with Uclust (command derep_fulllength) implemented in QIIME [25, 26]. Next, similar sequences (showing a percent identity of ≥ 99%) were clustered into one representative sequence with Uclust (command cluster_otus). Possible PCR chimeras, based on the ChimeraSlayer database of bacterial 16S rRNAs, were eliminated with Uchime implemented in QIIME [26, 27] and binned into “putative_chimera” The remaining sequences were subjected to local Blastn searches against bacterial 16S rRNA sequences of the Human Oral Microbiome Database (HOMD) [28] using the NCBI Blast plus program [29]. The top hit record (filtered by 99% identity) was applied as the species/taxonomic annotation of each representative sequence or categorized as a “blast no hit.” The species names and their read counts were normalized as a ratio (parts per million, ppm), and the ppm values were converted into logarithmic values. In the logarithmic conversion, populations of <1 ppm were defined as 0. These values were used for comparative diversity analyses, including principal component analysis (PCA), principal coordinate analysis (PCoA), α-diversity estimation, and multivariate analyses by using the programs R [30] and PAST [31].

## Results and Discussion

### Comparison of the obtained reads among the three DNA extraction methods

In the first sample set, a total 25 DNA samples were extracted from dental plaque from three volunteers using the three methods (Table 1). The number of average reads number obtained from the sequencer was 57,729 (standard deviation = 6,256). The number of average reads number passed that of the quality control (QC) standard (i.e., 56,452 ± 6,077). Most of the QC passed sequencing reads (79.5%) were successfully assigned to one of the reference bacterial 16S sequences contained in the HOMD database (S1 Table) [28] The remaining unpassed reads consist of potential chimera (5.9%), singletons (10.6%) and Operational Taxonomic Units (OTUs) without blast hit (4.0%). We excluded the singletons from following data analysis because of their low reliability. There was no statistically significant difference among obtained, QC passed, and assigned read number when subcategorized based on the person or the DNA extraction method (one-way ANOVA, p < 0.05).

### Estimation of oral flora from extracted DNA

**Fig 1:**
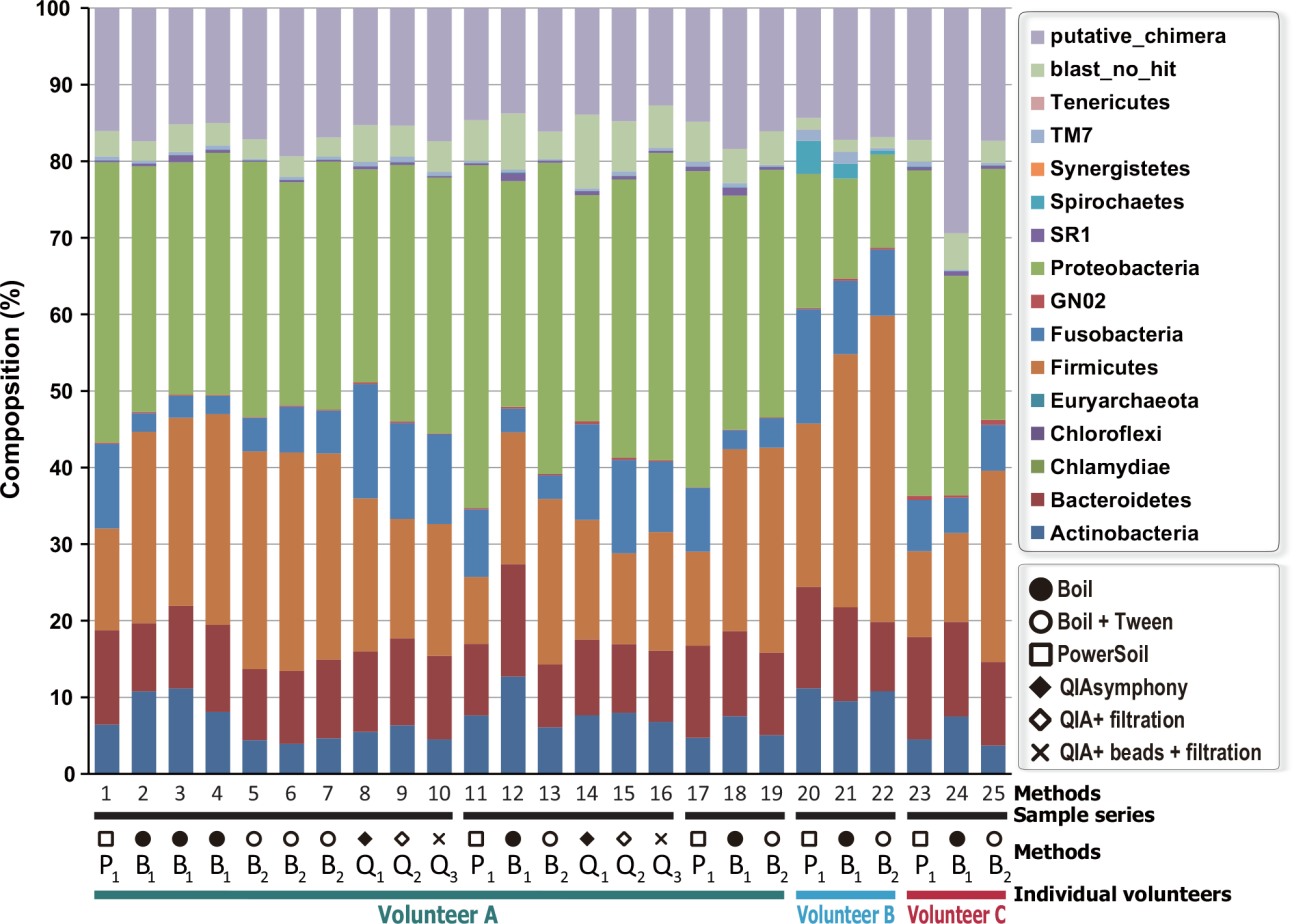
Distribution of microbes at the phylum level found in each of the 25 samples. Detailed information for the sample number is shown in Table 1. P1, B1, B2, Q1, Q2, and Q3 represent PowerSoil, boiling, boiling with tween, QIAsymphony, QIAsymphony with filtration, and QIAsymphony with beads beating and filtration, respectively.

The purpose of this study is a fine comparison of the DNA extraction methods rather than a fine characterization of each flora. Therefore, we chose species-level exact annotation provided by Blast rather than flexible one by QIIME which is more conservative and finishes in higher taxon in absence of highly homologous references [26]. In particular, we applied both OTU clustering at 99% instead of 97% and one-by-one assignment to the reference sequences in HOMD by Blastn. It was concerned that reads without annotation might be increased under the protocol; however, we observed only 4% in average owing to the high sequence accuracy by Miseq and completeness of the HOMD. With such a policy, each OTU were once assigned at species level then deduced to phylum level to get an overview of the flora (Fig 1). As a result, some differences were observed among the three methods of DNA extraction. For example, the population of Firmicutes tended to increase, whereas the population of Fusobacteria decreased using the boiling method compared with the other methods. On the other hand, the populations of these two phyla were similar using the PowerSoil and QIAsymphony DNA. However, reads of putative chimera from PCR artifacts (5.9% on average) were not statistically different among the three methods by one-way ANOVA. There were also some reads without homologues in the HOMD database (although a low percentage), implying that there are some undescribed bacteria in the human oral cavity.

### Reproducibility and completeness of each DNA extraction method

**Fig 2:**
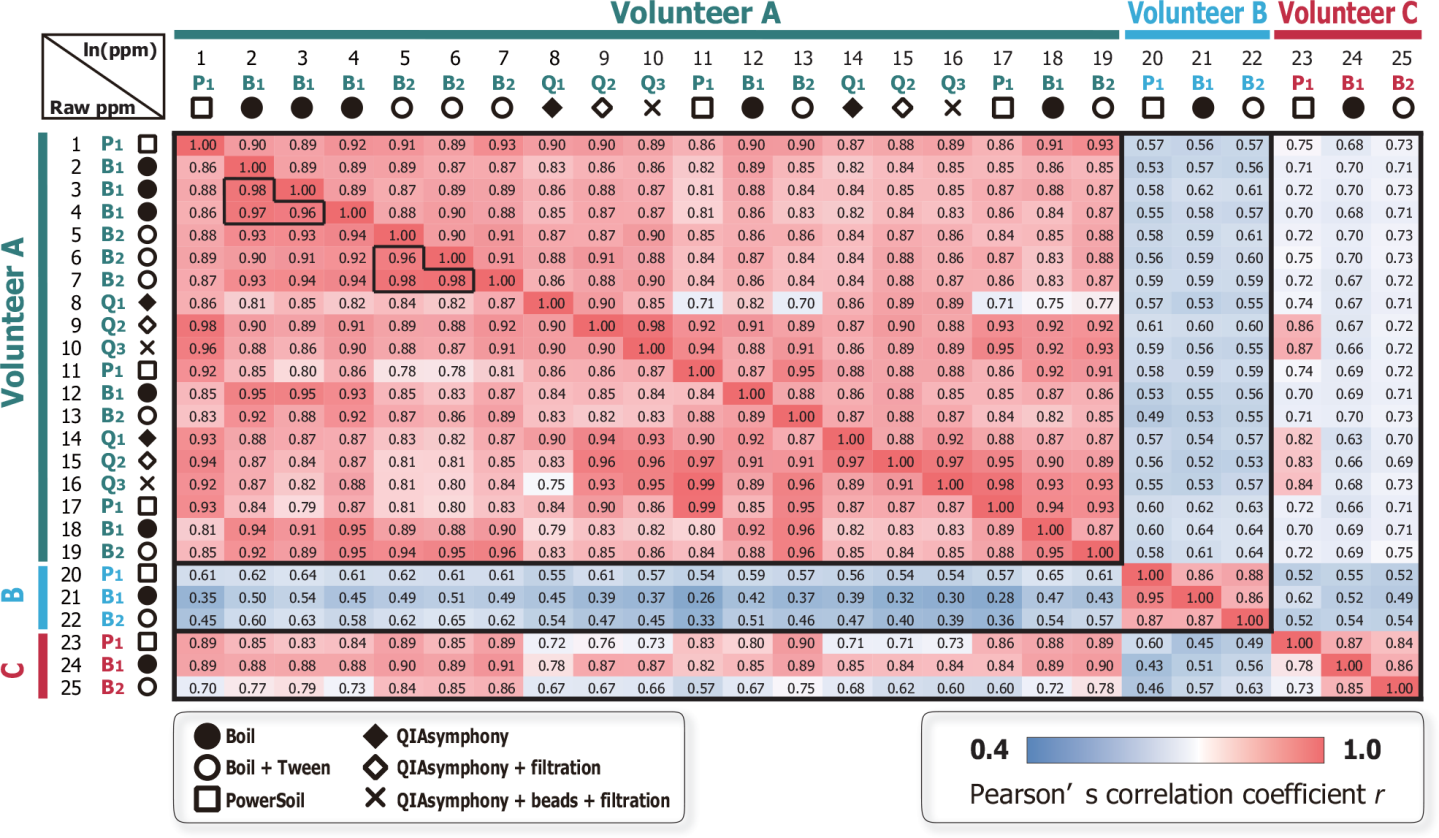
Correlation coefficient (*r*) matrix among assigned microbes for each sample. Upper right half represents correlation coefficients based on a logarithm-converted value. Lower left half represents those on linear value. Descriptions of each sample are shown in Table 1 and Fig 1.

To evaluate the reproducibility of the boiling method, DNA extraction was repeated three times and then assigned to one of the 688 entries in HOMD database. Using the boiling method alone or with tween, correlation coefficients (*r*) were high (0.96–0.98) between the triplicates as emphasized by a surrounding solid line (Fig 2). This suggests that the boiling method is reproducible.

The completeness of each DNA extraction method was also examined. Given that these purified DNA samples are to be used for metagenome analysis, it is critical to minimize the number of species we fail to detect. To evaluate such completeness, we calculated the α-diversities using the Shannon index, Simpson index, Chao-1 index, number of species >100 ppm, and number of species >1000 ppm (Table 2). We found no significant differences in α-diversity among the three methods, indicating that no single DNA extraction method has a clear disadvantage for completeness.

**Table 2:** Index of α-diversity for each DNA extraction method.

Shannon	PowerSoil	Boil	Boil +Tween	QS1	QS2	QS3
A-1	3.38	3.47	3.45	3.61	3.56	3.38
A-2	2.84	3.44	3.04	3.37	3.31	3.05
A-3	2.92	3.40	3.21	NA	NA	NA
B-1	4.13	4.04	3.91	NA	NA	NA
C-1	3.39	3.55	3.52	NA	NA	NA
Simpson Index (1-D)	PowerSoil	Boil	Boil +Tween	QS1	QS2	QS3
A-1	0.93	0.94	0.94	0.96	0.94	0.92
A-2	0.84	0.94	0.89	0.94	0.92	0.87
A-3	0.85	0.93	0.91	NA	NA	NA
B-1	0.97	0.97	0.97	NA	NA	NA
C-1	0.93	0.95	0.94	NA	NA	NA
Chao-1 Index	PowerSoil	Boil	Boil +Tween	QS1	QS2	QS3
A-1	186	180.33	169.7	183	193	182
A-2	174	176	170	183	187	163
A-3	196	186	179	NA	NA	NA
B-1	203	197	165	NA	NA	NA
C-1	181	169	191	NA	NA	NA
>100ppm	PowerSoil	Boil	Boil +Tween	QS1	QS2	QS3
A-1	114	119	116.3	125	137	114
A-2	104	111	100	117	122	101
A-3	106	128	114	NA	NA	NA
B-1	162	146	139	NA	NA	NA
C-1	127	109	122	NA	NA	NA
>1000ppm	PowerSoil	Boil	Boil +Tween	QS1	QS2	QS3
A-1	64	53.7	61	65	70	64
A-2	53	57	51	57	59	57
A-3	60	54	56	NA	NA	NA
B-1	97	86	84	NA	NA	NA
C-1	64	53	64	NA	NA	NA

A-1: sample ID 1–10 shown in Table 1. A-2: sample ID 11–16. A-3: sample ID 17–19. B-1: sample ID 20–22. C-1: sample ID 23–25. QS1: QIAsymphony. QS2: QIAsymphony + filtration. QS3: QIAsymphony + beads beating + filtration.

### Comparison of the sequencing results obtained from the three DNA extraction methods

**Fig 3:**
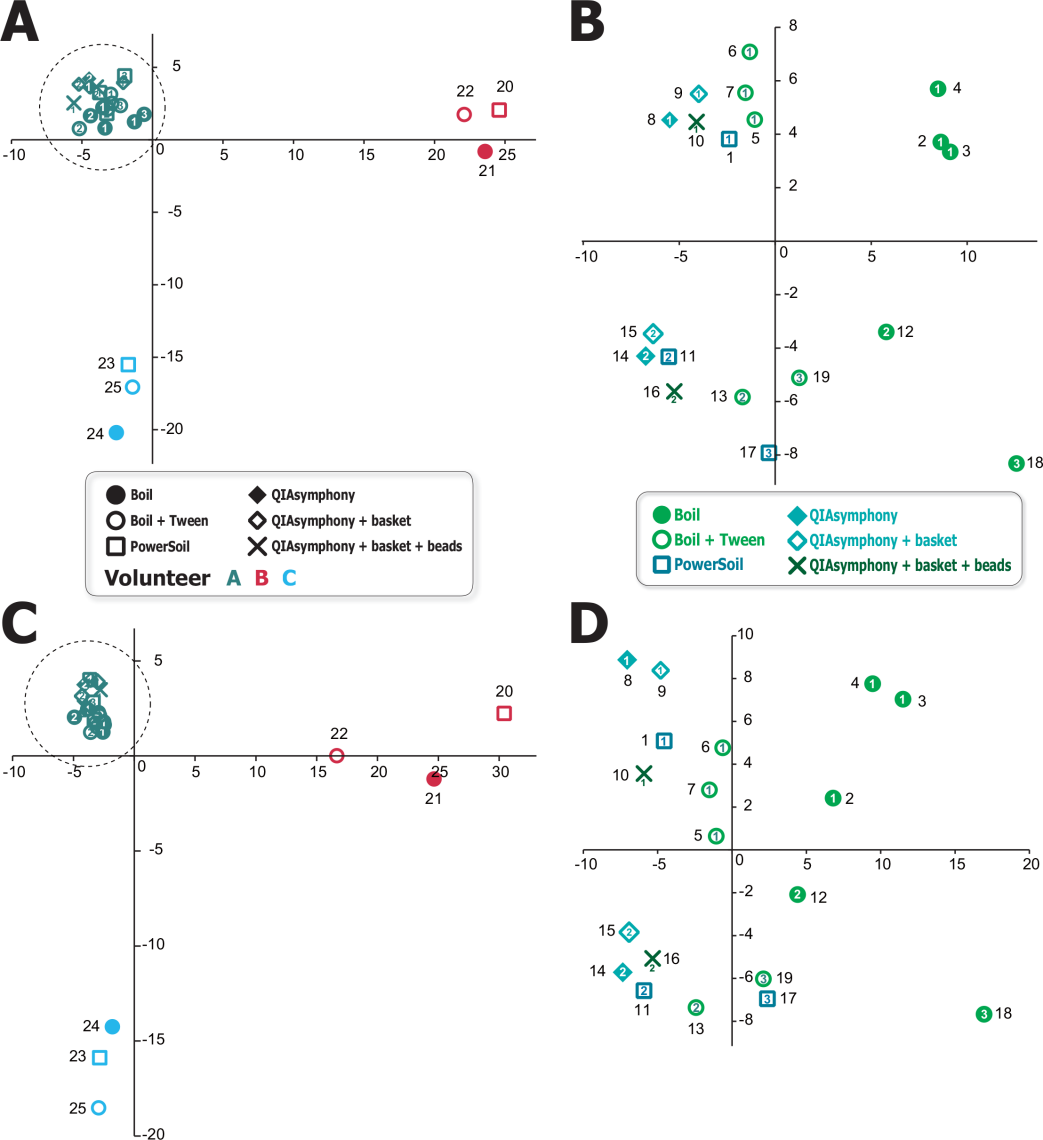
2D plot resulting from principal component analysis. A) Plot based on logarithm-converted values for the 25 samples. B) Plot based on the 19 samples from volunteer A enclosed by the dashed circle in panel A. C) Plot based on linear values for the 25 samples. D) Plot based on the 19 samples from volunteer A enclosed by the dashed circle in panel C. Horizontal and vertical axis represent PC1 and PC2, respectively. Sampling day numbers of volunteer A are shown as numerals.

To evaluate the similarity among the DNA extraction methods, correlation coefficients were calculated (Fig 2). We determined both the linear ppm based correlation coefficient and those based on the logarithm-converted value. The former is simpler but tends to ignore critical differences in small populations, whereas the latter can follow differences in small populations, although it is more qualitative. In both cases, it was clear that the correlation factors were higher among the same sample than among the same extraction methods. To further examine this, we performed PCA analysis (Fig 3), which indicated that data were clearly separated according to sample source (Fig 3A). Furthermore, when we focused on samples from volunteer A, they clustered according to the sample day (Fig 3B). Moreover, technical replicates formed tight clusters; for example, data obtained using the QIAsymphony method clustered tightly within the same sample source (volunteer A). Interestingly, data obtained using PowerSoil clustered closely with that of QIAsymphony, suggesting that PowerSoil and QIAsymphony give highly similar results. This is reasonable as both the methods are based on a silica matrix adsorption method combined with membranes or beads for DNA purification.

Compared with Fig 1, the differences among samples and methods are more obvious using PCA. This may be because of the fact that in PCA, data were categorized into 688 HOMD entries instead of the 17 phyla, and then the frequencies were converted into logarithmic values. Linear values tend to underestimate the contributions of small values. Indeed, when we executed PCA analysis with the linear ppm values (Fig 3C and 3D), the clusters became more obscure than when we used the logarithmic ones (Fig 3A and 3B).

In some previous studies comparing DNA extraction methods, considerable biases depending on the chosen method were observed [12, 14, 15]. However, consistent with our observation, other studies suggest that the results obtained from each DNA extraction methods are highly similar [10–12]. This discrepancy can be explained by differences in the DNA analysis method used and the samples obtained. Indeed, most previous studies that found biases used low-resolution methods in terms of taxonomic identifications, such as DEEG [11, 14]. By contrast, high-resolution NGS-based methods have only recently become available and were also used in this study [9, 12]. Moreover, in this study, we showed that logarithmic conversion improves the resolution in species identification and clustering analyses. In addition, some previous studies that found biases used artificial flora [9, 15]. In such cases, it is possible to compare the absolute microbial population to that obtained from the experiment, and therefore determine whether any bias exists. Although this seems to be an ideal situation, a clear drawback is that these biases are only applicable for those particular microbes that were examined. Strictly speaking, the same species-assignment may be potentially diversified if there are differences in the strain and/or genotype. Moreover, it is virtually impossible to examine an artificial flora that is consistent with all the microbes found worldwide. Therefore, in this study, we examined natural bacterial flora for evaluating the biases. The advantage of this strategy is that we can evaluate many more species than the artificial one. However, the con is that it is impossible to know the true bacterial composition in the flora, making it difficult to evaluate each method. Nevertheless, we demonstrated that the differences among the three methods were considerably smaller than the differences in the three samples.

Altogether, these observations suggest that the three DNA extraction methods give comparable results and can potentially be used to differentiate the individual who provides the samples. In addition, we showed that logarithm conversion endowed more resolution for these techniques.

### Optimization of the boiling method

Faced with metagenomic studies requiring a large number of samples, the time and financial cost for DNA extraction become important factors. As shown above, the boiling method has no critical disadvantage compared with the other techniques. Based on its low cost, we further optimized this method and performed a detailed analysis with another larger set of plaque samples (n = 12). In the second sample set, a total of 72 DNA samples from dental plaque were obtained from 12 volunteers and using the following six methods: PowerSoil, boiling, boiling with bead beating, tween, mutanolysin, or lysostaphin treatment. The number of average reads number obtained from the sequencer was 121,322 ± 25,973, whereas the number of average reads number that passed the QC standard was 117,862 ± 25,369. Most of these reads (69.3%) were successfully assigned to one of the reference bacterial 16S sequence contained in HOMD database (S1 Table). There were no statistically significant differences among reads obtained, QC passed, and assigned read number when subcategorized based on DNA extraction method (one-way ANOVA, p < 0.05).

**Fig 4:**
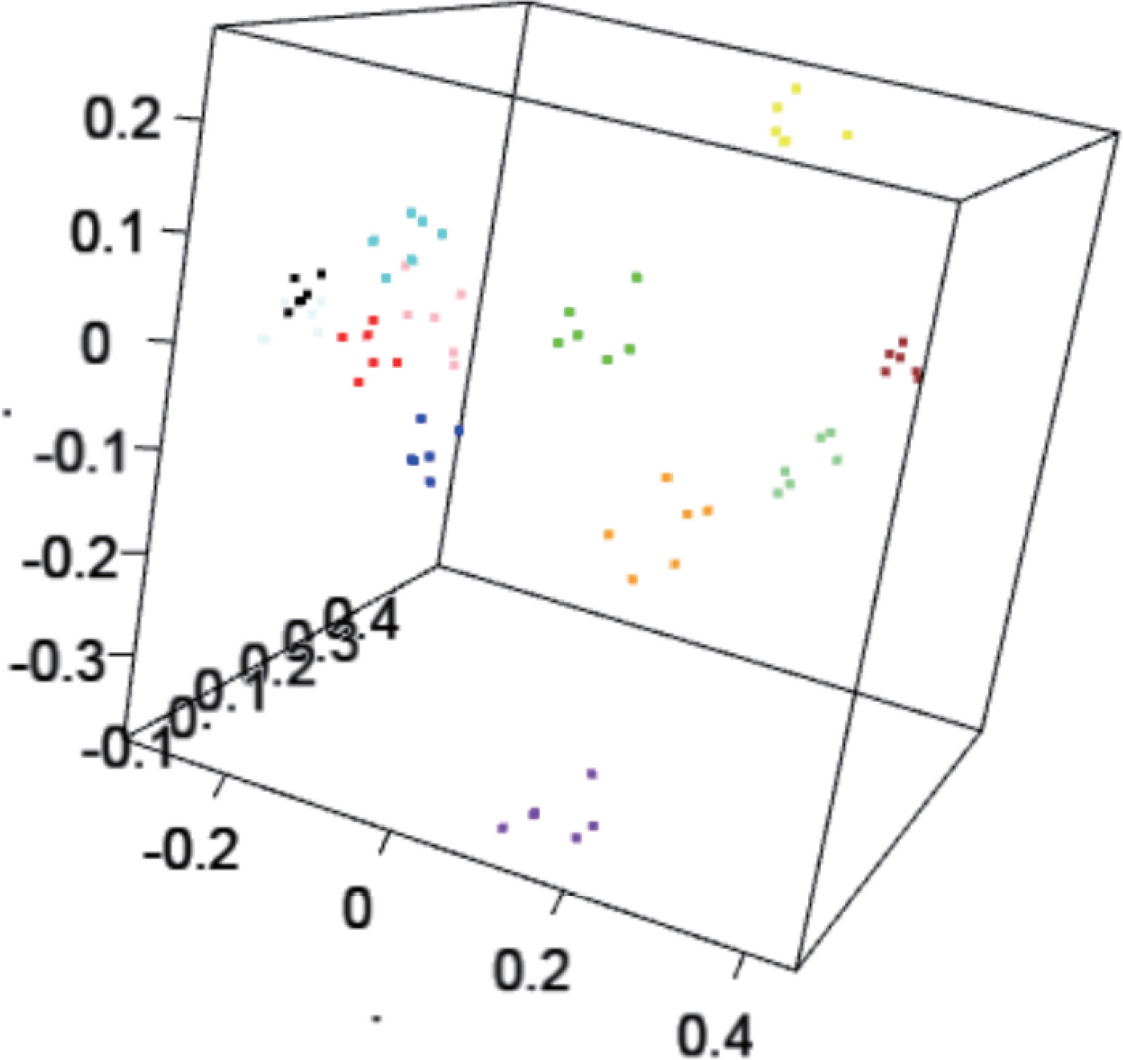
3D plot resulting from principal coordinate analysis derived from 12 samples and the six different boiling methods. Dots with respective colors indicate they are from the same sample.

Using PCoA analysis, 12 obvious clusters appeared to correspond to the 12 individual volunteers, consisting of six points that correspond to each DNA extraction method (Fig 4). This observation suggests that the observed variations derived from methodological differences were smaller than those from the individual sample flora, in concordance with smaller sample set described above.

### Minor differences among the DNA extraction methods

Despite the concordance among the DNA extraction methods, the assigned OTUs derived from each method were not identical. To determine whether there were any specific OTUs whose extraction efficiency was high or low compared with others, we examined deviation by the methods in each OTU and each specimen whether it was five times more than the median (high extraction efficiency group) or was one fifth times less than the median (low extraction efficiency group). To qualify the data, OTUs were exempted from the analysis if all of the read popuration by each DNA extraction methods in an OTU were less than 100 ppm for high extraction efficiency group or if median was less than 100 ppm for low extraction efficiency group. The number of high/low cases from the 12 samples was tallied for each OTU (Table 3, S2 Table). To find any correlations between the observed biases in DNA extraction and biological feature of microbes, an enrichment analysis based on cell wall structure (gram positive or negative) was performed. Firstly, we selected constantly detected 166 OTUs over the 12 specimens (S2 Table). Among them, 71 belonged to gram positive microbes and 95 belonged to gram negative one. On the other hand, DNA extraction efficacy was positively biased in 19 OTUs with the boil and beads beating method out of the 166 OTUs. Among them, 14 belonged to gram positive microbes and five belonged to gram negative one. We executed Fisher's exact test and found beads beating tended to improve DNA extraction efficacy on gram positive microbes (p-value = 0.0057). On the other hand, the remaining biological features such as spore formation had not been tested with a paucity of encyclopedic biological annotation for bacteria, like gene ontology.

**Table 3:** Variation of DNA extraction efficiency using each method.

A. List of OTU extracted with the hightst efficiency						
	boil	boil + Beads	boil + Tween20	boil + Lysostaphin	boil + mutanolysin	PowerSoil
HOMD ID 008: Mogibacterium vescum	1/6	6/6	0/6	0/6	0/6	0/6
HOMD ID 807: Olsenella sp._	1/6	4/6	0/6	0/6	0/6	0/6
HOMD ID 909: Leptotrichia sp.	0/7	3/7	0/7	1/7	4/7	1/7
HOMD ID 498: Leptotrichia sp.	0/9	2/9	0/9	0/9	5/9	0/9
HOMD ID 100: Lachnospiraceae sp.	0/10	5/10	2/10	0/10	0/10	0/10
HOMD ID 112: Peptostreptococcus stomatis	1/8	4/8	0/8	0/8	1/8	0/8
HOMD ID 170: Actinomyces sp.	0/12	6/12	0/12	1/12	1/12	0/12
HOMD ID 278: Porphyromonas sp.	0/6	3/6	3/6	0/6	1/6	2/6
HOMD ID 557: Eubacterium brachy	1/8	4/8	0/8	1/8	1/8	0/8
HOMD ID 313: Prevotella sp.	3/6	2/6	0/6	1/6	0/6	0/6
HOMD ID 414: Actinomyces sp.	3/6	2/6	0/6	0/6	0/6	1/6
HOMD ID 278: Porphyromonas sp.	0/6	3/6	3/6	0/6	1/6	2/6
B. List of OTU extracted with the lowest efficiency						
	boil	boil + Beads	boil + Tween20	boil + Lysostaphin	boil + mutanolysin	PowerSoil
HOMD ID 563: Leptotrichia buccalis	5/8	0/8	0/8	3/8	0/8	1/8
HOMD ID 758: Streptococcus sanguinis	7/12	0/12	0/12	2/12	2/12	1/12
HOMD ID 498: Leptotrichia sp.	4/7	0/7	4/7	1/7	0/7	1/7
HOMD ID 666: Corynebacterium matruchotii_	0/12	0/12	0/12	5/12	6/12	0/12
HOMD ID 056: Streptococcus sp.	1/12	0/12	0/12	1/12	3/12	6/12
HOMD ID 178: Actinomyces sp.	0/10	0/10	0/10	1/10	1/10	5/10

The counts represents the number of biased cases in each microbe with each extraction method. The denominators correspond to the number of spcimens given by the 12 volunteers which contained qualified popuration of each microbes. The definitions of the biased case and the qualified popuration are described in the main text. Representative OTUs with significant biases are shown. For all OTU data, see S2 Table.

Among the six methods, boiling with bead beating showed a slightly better performance than the others, consistent with some previous studies [9–11]. However, the bead beating method tended to show low performance for detecting *Enterococcus italicus* (HOMD ID 803), TM7[G-1] sp. (HOMD ID 347) and TM7[G-1] sp. (HOMD ID 353) as an example (S2 Table). Despite this, it is possible that the different genotypes may affect the effectiveness of DNA extraction even for bacterial strains in the same category. This suggests that there is no “gold standard” in DNA extraction method that shows the best performance in all situations. Instead, it is important to select the best method according to the specimen and aim of each study. For example, in oral metagenomics, *Streptococcus mutans* and red complex (involved in caries and periodontal disease, respectively), were species that were never missed in our study by all three methods. Therefore, all three methods we examined fulfilled the demands of a 16S metagenomics for an oral specimen. Although the bead beating method demonstrated the best performance in this study, it only showed a minimal advantage. Therefore, the choice of the DNA extraction method should be balanced with the additional cost and effort, especially in a passive parallel analysis with NGS.

## Conclusion

We evaluated three different DNA extraction methods for human oral plaques, each with their own advantages. Among them, the boiling method is not only superior in its simplicity, cost, and short handling time, but it is also reliable, based on its low bias. Performance of the boiling method has been also demonstrated for fecal microbes [12], suggesting that it could be widely feasible for other microbes and specimens. PowerSoil, one of the most famous methods in this arenas, and QIAsymphony gave almost identical results, suggesting that the QIAsymphony is a promising alternative in automation. On the other hand, a more detailed analysis at the species level demonstrated that each method has its preferred species in terms of efficiency of DNA extraction. Nevertheless, these can be regarded as exceptions in terms of the wider bacterial population. In conclusion, there is no one “gold standard” method that should be used for DNA extraction for use in NGS. Moreover, the biases in DNA extraction do not have to be overvalued as long as there is no bias in the set of microbes of specific interest for your study. Focusing on the boiling method, it is still unknown whether the extracted DNA is stable for long storage; however, it was demonstrated that it was enough feasible at least for a one-time sample preparation for 16S DNA metagenome analysis. With the increasing performance of NGS, DNA extraction will remain a “bottle neck” for data production. This is also true for clinical sites where rapid diagnosis is required. In this respect, the boiling method described here may offer a potential solution for the DNA extraction step in this field.

## Supporting Information

S1 Table Mapped reads for each Human Oral Microbiome Database (HOMD) entry. (XLSX)

S2 Table Observed bias among the DNA extraction methods for all Operational Taxonomic Unit (OTUs). The number of volunteers (out of the 12 total examined) that showed high or low extraction efficiency for each OTU is shown. The definitions of high and low extraction efficiency are described in the main text.
(XLSX)
